# Correlation of Dental and Periodontal Status With HIV Presence and Initial CD4 Counts: An Albanian Prospective Observational Study

**DOI:** 10.7759/cureus.76419

**Published:** 2024-12-26

**Authors:** Eriselda Simoni (Malushi), Leonard Simoni, Laureta Flaga, Arjan Harxhi, Najada Como

**Affiliations:** 1 Oral and Maxillofacial Surgery, University Dental Clinic, Tirana, ALB; 2 Cardiology, University Hospital Center “Mother Teresa”, Tirana, ALB; 3 Public Health, Family Medicine, Health Center 5, Tirana, ALB; 4 Infectious Diseases, University Hospital Center “Mother Teresa”, Tirana, ALB; 5 Infectious Diseases, Faculty of Medicine, University of Medicine, Tirana, ALB

**Keywords:** cd4 counts, community periodontal index (cpi), decayed missing and filled teeth (dmft), human immunodeficiency virus (hiv) infection, loss of attachment (loa)

## Abstract

Background

Different pathologies are encountered more often in human immunodeficiency virus (HIV)-infected patients, such as bacterial, fungal, viral infection, and neoplastic diseases. Recently, studies have shown that HIV-infected individuals have poorer oral health outcomes, worse dentition, and aggressive forms of periodontitis. This study aims to investigate the dental and periodontal status of HIV-infected patients, the correlation between CD4+ level and the CD4 percentage with dentition, and periodontal status.

Methodology

A prospective observational study was conducted in the University Dental Clinic and the Infective Service of University Hospital Center “Mother Teresa” in Tirana, Albania. All patients newly diagnosed with HIV infection (35 patients, 40.7%) and those without HIV infection (51 patients, 59.3%) who underwent oral examination from April through July 2024 were included. Patients were grouped according to HIV status into two groups. This study evaluated the basic demographic characteristics, laboratory measurements, especially CD4 counts, oral hygiene, and the presence of dental and periodontal lesions. The dentition status was assessed using the values of decay teeth (DT), filled teeth (FT), and missing teeth (MT), presented as DMFT. The periodontal status was evaluated through a periodontal probe measuring community periodontal index (CPI) and loss of attachment (LOA), as recommended by the World Health Organization’s Oral Health Assessment Form 2013. The Pearson’s correlation coefficient (r) was used to evaluate the correlation between the levels of CD4+ and DMFT, CD4+ and CPI, CD4+ and LOA, CD4+/lymphocyte percentage (CD4%) and DMFT, CD4% and CPI, and CD4% and LOA. P-values ≤0.05 were considered statistically significant.

Results

HIV-infected patients had a worse dentition status, with higher DT, higher MT, and higher DMFT index values (9.71 ± 6.72 vs. 5.96 ± 4.49, p = 0.003) compared to those without HIV. HIV-infected patients also had a worse periodontal status, with higher CPI (2.63 ± 1.06 vs. 0.94 ± 0.68) and LOA (2.57 ± 1.06 vs. 0.94 ± 0.68) compared to those without HIV. An important negative correlation was found between CD4+ and dentition and periodontal status. A lower level of CD4+ was correlated with a higher DMFT (r = -0.52, p = 0.01, CPI (r = -0.38, p = 0.024, and LOA (r = -0.37, p = 0.029).

Conclusions

HIV-infected patients manifest a worse dentition and periodontal status, with the worsening strongly correlated with the initial CD4+ levels. Periodontal disease may serve as a significant clinical indicator for the early diagnosis of HIV and its progression. Dental professionals should be vigilant in assessing periodontal health, especially in high-risk populations, as it may prompt timely testing and intervention for HIV infection.

## Introduction

Human immunodeficiency virus (HIV) is a virus that attacks the immune system, specifically targeting CD4+ T cells. If untreated, it can lead to the progressive deterioration of the immune system [1]. Acquired immunodeficiency syndrome (AIDS) presents the most severe stage of HIV infection, characterized by a significantly weakened immune system and the occurrence of specific opportunistic infections, cancers, or other severe clinical manifestations. A diagnosis of AIDS is made when the CD4+ T-cell count falls below 200 cells/mm³ or when certain opportunistic infections or cancers arise [1]. Since the beginning of the epidemic, about 36 million people have died from HIV/AIDS-related illnesses. Antiretroviral therapy has significantly reduced mortality rates, transforming HIV from a fatal disease to a manageable chronic condition [1]. Albania has a low HIV prevalence epidemic, but, in recent years, the number of HIV cases has increased. From 1993 to 2023, 1,716 cases were diagnosed, and around 60% of new cases were diagnosed in the late stages of the infection [2].

Individuals with HIV/AIDS often experience various oral health issues, including oral thrush, candidiasis, periodontal diseases, and lesions such as Kaposi sarcoma [3]. The most common manifestations of periodontal pathology are linear gingival erythema, necrotizing ulcerative gingivitis, and necrotizing ulcerative periodontitis [3]. These conditions can lead to discomfort, difficulty in eating, and impaired quality of life [4]. The immune system compromise associated with HIV/AIDS can exacerbate periodontal disease. Studies have shown that individuals with HIV are associated with poorer oral health outcomes, worse dentition, and are more prone to aggressive forms of periodontitis, which can lead to gum recession, tooth mobility, and tooth loss [5-7]. Early recognition of these oral manifestations is vital for prompt HIV testing and diagnosis, as well as the initiation of antiretroviral therapy, which can significantly improve patient outcomes [8]. This study aims to investigate HIV-infected patients’ dental and periodontal status, the correlation between CD4+ level and the CD4 percentage with dentition, and periodontal parameters.

## Materials and methods

A prospective observational study was conducted in the University Dental Clinic and the Infective Service of University Hospital Center “Mother Teresa” in Tirana, Albania. All patients newly diagnosed with HIV and those without HIV who underwent oral examination from April through July 2024 were included. The study complied with the Declaration of Helsinki and was approved by the Tirana University of Medicine Ethics Committee (approval number: 11, dated 18.03.2024). Informed consent was obtained from every patient who underwent a dental examination. This is one of the first studies conducted in Albania on this subject.

Patients were grouped according to HIV status into two groups. Group 1 included patients with known HIV infection (HIV group). Group 2 included patients without known HIV infection (W-HIV group). Group 1 included only newly diagnosed HIV-infected patients who were not yet under antiretroviral therapy. All patients under 15 years old were excluded from the study.

Information about demographics, comorbidities, and clinical history were collected. Specifically, for HIV patients, the levels of CD4+ and CD4/lymphocyte percentage were assessed, and based on them, the disease stages of each patient during medical examination were collected using a structured questionnaire. We used the World Health Organization (WHO) and the European Association of Dental Public Health criteria for evaluating oral health status [9].

We measured clinical loss of attachment (LOA), probing pocket depth, and tooth mobility to evaluate periodontal disease. Clinical LOA measures the extent of periodontal support lost around a tooth. It reflects the position of the soft tissue related to the cementoenamel junction (CEJ), a fixed landmark on a tooth. To calculate LOA we determined two main measurements, namely, the probing depth (PD) and the gingival recession (GR), or the distance from the gingival margin (GM) to the CEJ [10].

The PD was calculated by measuring the depth at which a periodontal probe penetrates the tissue below the sulcus. Healthy gingiva typically measures between 2.0 mm and 3.0 mm. The GR was calculated by measuring the distance from the CEJ to the gingival margin, noting the location of the last relative to the CEJ. On the assessment of the position of the gingival margin relative to the CEJ, the values can be positive, negative, or 0 mm at the CEJ level. This distance was added to the PD. Once the necessary measurements were obtained, we calculated LOA using the following formula: clinical LOA (mm) = PD (mm) + GR (mm) [9]. However, the result was based on the positioning of the gingival margin as follows: apical to the CEJ: LOA = PD + GR; at the level of the CEJ: LOA = PD + 0 mm; and coronal to the CEJ (recession): the adjusted recession value of LOA = PD - GR.

These measurements are critical for understanding and monitoring changes in periodontal support, ensuring accurate and consistent clinical evaluations. A higher LOA indicates greater periodontal tissue loss, which is essential in assessing the severity and progression of periodontal disease. Correct measurement and interpretation are crucial for effective diagnosis and treatment planning.

The periodontal status was also evaluated using a periodontal probe measuring community periodontal index (CPI), as recommended by the WHO Oral Health Assessment Form 2013. The periodontal status was assessed by probing the teeth numbered 11, 16, 17, 26, 27, 31, 36, 37, 46, and 47, evaluating the presence of gingival bleeding, presence of supra- and subgingival calculus, periodontal pockets with PDs between 3.5 and 6.0 mm. According to this evaluation, the CPI is 0 in healthy gingiva, 1 in gingival bleeding, 2 in the presence of calculus, 3 for periodontal pockets measuring 4 to 5 mm, and 4 for pockets measuring 6 mm or more [11].

The number of decayed, missing, and filled teeth (DMFT) was determined. The dentition status was assessed by summarizing the values of decayed teeth, filled teeth, and missing teeth for each patient. For each group of patients (HIV and W-HIV), the overall DMFT was calculated as the sum of all individual DMFT values divided by the number of patients.

The main study endpoints included the differences in CPI, LOA, and DMFT values between HIV and W-HIV groups. Other important study endpoints were the correlation between the levels of CD4+ and DMFT, CD4+ and CPI, CD4+ and LOA, CD4+/lymphocyte percentage (CD4%) and DMFT, CD4% and CPI, and CD4% and LOA.

Statistical analysis

All categorical variables were presented as absolute numbers and percentages and compared using the chi-square test. Meanwhile, all continuous variables were presented as mean ± standard deviations, and Student’s t-test was used to calculate the differences between groups.

The Pearson’s correlation coefficient (r) was used to evaluate the relationship between the levels of CD4+ and DMFT, CD4+ and CPI, and CD4+ and LOA, as well as to assess the relationship between CD4% and DMFT, CD4% and CPI, and CD4% and LOA. P-values ≤0.05 were considered statistically significant. The statistical analysis was performed using SPSS Version 21.0 (IBM Corp., Armonk, NY, USA). 

## Results

Of the 86 patients who underwent oral examination, 35 (40.7 %) were included in the HIV-infected group, and 51 (59.3%) were in the without HIV-infected group (W-HIV). Table 1 shows the baseline data. The HIV-infected patients were mainly males (80.0% vs. 41.2%, p < 0.001). There were no differences between the groups relating to comorbidities. HIV-infected patients experienced more weight loss (25.7% vs. 0%, p < 0.001) compared to W-HIV and had worse oral hygiene (use of toothbrush <1 time a day) (57.1% vs. 9.8% p < 0.001).

**Table 1:** Characteristics of patients included in the study. *: To determine statistical significance for the comparison regarding each characteristic, variables were summarized using mean ± SD for continuous variables compared using t-tests and frequency and percentage for categorical variables compared using chi-squared tests. SD = standard deviation; CV = cardiovascular

Variables	HIV (n = 35)	Without HIV (n = 51)	P-value*
Gender male, n (%)	28 (80.00)	21 (41.17)	<0.001
Age mean (SD)	35.34 (12.60)	31.76 (11.81)	0.183
CV diseases, n (%)	3 (8.6)	8 (15.7)	0.332
Diabetes mellitus, n (%)	2 (5.7)	5 (9.8)	0.496
Arterial hypertension	3 (8.6)	9(17.6)	0.233
Impaired renal function, n (%)	1 (2.8)	0	0.225
Weight loss, n (%)	9 (25.7)	0	<0.001
Teeth brush use/day <1, n (%)	20 (57.14)	5 (9.80)	<0.001
Teeth brush/day = 1, n (%)	7 (20.00)	13 (25.49)	0.554
Teeth brush/day >1, n (%)	9 (25.71)	31 (60.78)	0.001

Characteristics of HIV patients

The characteristics of HIV-infected patients are shown in Table 2. The main HIV infection mode of transmission was through heterosexual contact (68.6%). The majority of patients examined were categorized in stage 3 (57.3%). The mean CD4+ level was 266.2 cells/m^3^ (normal range = 500-1,500 cells/mm^3^) and the CD4/lymphocyte mean percentage was 15.7% (normal range >26%).

**Table 2:** Characteristics of HIV patients. HIV = human immunodeficiency virus; AIDS = acquired immunodeficiency syndrome

HIV/AIDS patients (n = 35)	
Mode of transmission	
Heterosexual, n (%)	24 (68.6%)
Homosexual, n (%)	6 (17.1%)
Blood transfusion, n (%)	2 (5.7%)
Drug injections, n (%)	1 (2.9%)
Unknown, n (%)	2 (5.7%)
Clinical stage of HIV	
Stage I, n (%)	3 (8.6%)
Stage II, n (%)	12 (34.3%)
Stage III/AIDS, n (%)	20 (57.3%)
CD4 level cell/mm^3^ (mean)	266.2
CD4% (mean)	15.7
Periodontal disease	11 (31.4%)

Dental and periodontal status of patients

The dental and periodontal status of the study participants is shown in Table 3. HIV-infected patients had a worse dentition status, with higher DT (2.26 ± 3.05 vs. 0.39 ± 0.92, p = 0.0001), MT (2.77 ± 3.36 vs. 1.18 ± 2.54, p = 0.014) and DMFT index (9.71 ± 6.72 vs. 5.96 ± 4.49, p = 0.003) compared to W-HIV patients. Further, HIV-infected patients had a worse periodontal status, with higher CPI (2.63 ± 1.06 vs. 0.94 ± 0.68) and LOA (2.57 ± 1.06 vs. 0.94 ± 0.68) compared to W-HIV patients.

**Table 3:** Dental and periodontal parameters of patients. *: To determine statistical significance for the comparison regarding each one of dental and periodontal parameters, variables were summarized using mean ± SD for continuous variables compared using t-tests. DT = decay teeth; FT = filled teeth; MT = missing teeth; DMFT = decayed, filled, and missing teeth; CPI = community periodontal index;  LOA = loss of attachment; SD = standard deviation

Variables	HIV (n = 35)	Without HIV (n = 51)	P-value*
DT mean (SD)	2.26 (3.05)	0.39 (0.92)	<0.001
FT mean (SD)	4.69 (4.82)	4.39 (3.92)	0.757
MT mean (SD)	2.77 (3.36)	1.18 (2.54)	0.014
DMFT mean (SD)	9.71 (6.72)	5.96 (4.49)	0.003
CPI mean (SD)	2.63 (1.06)	0.94 (0.68)	<0.001
LOA mean (SD)	2.57 (1.07)	0.94 (0.68)	<0.001

Correlation between first CD4 level and DMFT, CPI, and LOA

**Figure 1:**
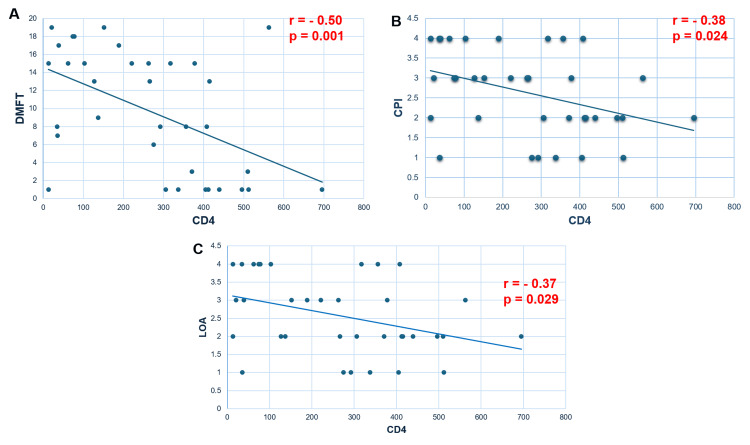
Correlation between first CD4 level and DMFT (A), CPI (B), and LOA (C). A. Scatterplot of data points of DMFT and CD4 variables with a negative Pearson’s correlation coefficient (r). The line is a negative slope. As one variable (CD4) increases, the other decreases (DMFT), with a strong negative linear correlation. B. Scatterplot of data points of DMFT and CPI variables with a negative Pearson’s correlation coefficient (r). The line is a negative slope. As one variable (CD4) increases, the other decreases (CPI). C. Scatterplot of data points of DMFT and LOA variables with a negative Pearson’s correlation coefficient (r). The line is a negative slope. As one variable (CD4) increases, the other decreases (LOA). DMFT = decayed, filled, and missing teeth; CPI = community periodontal index; LOA = loss of attachment

There was a significant negative correlation between the first CD4 level and DMFT (r = -0.50, p = 0.001), CPI (r = -0.38, p = 0.024), and LOA (r = -0.37, p = 0.029). The decrease of the first CD4 level correlated with the worsening of dentition and periodontal status with the increase in DMFT, CPI, and LOA (Figure 1).

**Figure 2:**
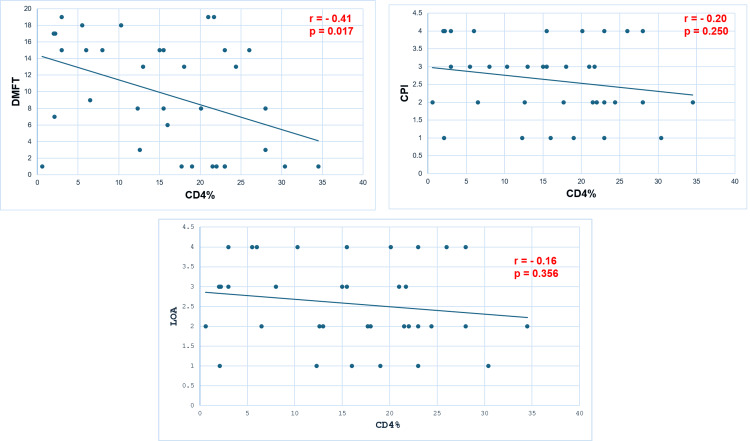
Correlation between CD4% and DMFT (A), CPI (B), and LOA (C). A. Scatterplot of data points of DMFT and CD4% variables. The line of best fit is a negative slope. As one variable (CD4) increases, the other decreases (DMFT), with a negative linear correlation. B. Scatterplot of data points of DMFT and CPI variables. The line of best fit is a quasi-horizontal slope, with a zero correlation. C. Scatterplot of data points of DMFT and LOA variables. The line of best fit is a quasi-horizontal slope, with a zero correlation. DMFT = decayed, filled, and missing teeth; CPI = community periodontal index; LOA = loss of attachment

There was a significant negative correlation between CD4 percentage level and DMFT (r = -0.41, p = 0.017). Moreover, there was a non-significant negative correlation between CD4% and CPI (r = -0.20, p = 0.25) and LOA (r = -19, p = 0.29). The decrease in the first CD4 level correlated with the worsening of dentition status (DMFT) but not with periodontal status (CPI and LOA) (Figure 2).

## Discussion

In the present study, we investigated the dental and periodontal status of HIV patients, and the correlation between the first CD4 level and CD4% with the oral health, dentition, and periodontal status. We documented that the HIV patients had (a) significantly worse dentition status and higher number of DT, MT, and DMFT index compared to W-HIV; (b) a worse periodontal status, with higher CPI and LOA compared to W-HIV; and (c) reduced first CD4 level correlated with the worsening of dentition and periodontal status with the increase in DMFT, CPI, and LOA.

In our study, periodontal disease was noted in 31.4% of HIV-infected patients. Many studies demonstrate that 50-90% of HIV-positive patients have oral manifestations, increasing according to HIV/AIDS stages [7] Patton et al. [6] showed that 48% of HIV/AIDS patients had at least one oral manifestation. The study found that the main manifestations were hairy leukoplakia (26.5%), candidiasis (20%), and HIV-associated periodontal diseases (8.8%). Margiotta et al. [12] in an Italian population found that CD4+ cell counts <200 × 10^6^/L were significantly associated only with strongly associated lesions such as hairy leukoplakia and periodontal diseases (p = 0.03). The median values of CD4+ cell count were also correlated significantly (p = 0.02). Another study conducted in South Africa by Arendorf et al. [13] demonstrated that 60.4% of patients had at least one oral lesion, with 37.8% candidosis, 19.7% oral hairy leukoplakia, and 8.5% HIV-related periodontal disease.

Patients with HIV infection had a worse dentition status, with higher DT, MT, and DMFT index compared to W-HIV patients (DMFT = 9.71 ± 6.72 vs. 5.96 ± 4.49, p = 0.003). This status was worse in the advanced stages of the disease, as expressed by the first CD4+ level and CD4%. Congruent to our findings, Kumar et al. [14] documented that the mean DMFT score was significantly higher in HIV-positive individuals (12.83 ± 9.6) compared to HIV-negative (8.34 ± 7.6) patients (p < 0.0001). In another study, De Lima et al. [15] observed higher mean DMFT values in HIV individuals than in the control group (10.07 vs. 8.94). In a study conducted in Iran [16], the mean value of the DMFT index of HIV/AIDS patients was found to be 11.87 ± 8.08. On the contrary, in some studies, DMFT did not differ significantly between HIV and general dental patients [17].

Caries is considered one of the most common oral diseases in HIV-positive patients, with a reported prevalence ranging between 54% and 83% [3]. Different factors are associated with caries in HIV/AIDS patients, who have worsened dental hygiene. In our study, we found that brushing less than one time a day was significantly higher in HIV-infected patients compared to W-HIV (57.1% vs. 9.8% p < 0.001). Access to dental care can be a challenge for individuals with HIV infection, often due to stigma, financial constraints, or a lack of awareness about the importance of oral health. This can lead to untreated oral diseases, compounding health issues. HIV can affect salivary flow and composition, leading to xerostomia (dry mouth). Reduced saliva can increase the risk of dental caries and oral infections, further complicating oral health [3]. Furthermore, the predisposition to dental caries increases significantly with age due to the continuum effect of viral infection, immune and anti-inflammatory response, concomitant diseases, and duration of antiretroviral therapy [8].

In our study, we documented a worse periodontal status in HIV/AIDS, higher CPI, and LOA compared to W-HIV/AIDS, and this status was worse in the advanced stages of the disease, expressed in the first CD4+ level and CD4%. Our findings are in line with other studies. Kumar et al. [14] showed a significant difference in the CPI and LOA between HIV-positive and control groups. Half of the HIV patients had CPI and LOA scores greater than 2. Pereira et al. [18] documented that infected individuals were five times more likely to have periodontitis than those without HIV (ORA = 5.554 (1.596−19.324), p = 0.007). McKaig et al. [7] documented that HIV-infected persons from North Carolina presented severe findings. Overall, 62% of persons had a probing PD ≥5 mm, 46% had recession ≥3 mm, and 66% had attachment level ≥5 mm in one or more sites. Another study by Berniyant et al. [19] documented that HIV/AIDS individuals had a poor periodontal status due to a higher number of bleeding on probing, periodontal PD, and LOA, even though only the LOA difference between groups was not statistically significant. A recent study conducted in Uganda [20] among HIV patients found that 66% of the individuals had probe bleeding, and around half had moderate-to-severe clinical attachment loss. Similar to our study, Pattrapornnan et al. [21] in a study conducted among HIV-positive pregnant women found an important association between Low CD4 levels and periodontitis (OR = 2.06, 95% CI = 1.00-4.27, p = 0.05).

There are different pathophysiological pathways involved in the development of periodontal disease in HIV/AIDS patients. The progression of periodontal disease in HIV-positive individuals is often linked to a decline in immune function [8]. As the immune system weakens, the body becomes less capable of controlling bacterial infections in the gums, leading to more severe periodontal issues due to tissue inflammation, as well as the destruction of teeth surroundings and support [6,7,14-16]. Periodontal disease can serve as an important indicator for the early diagnosis of HIV and its progression. The presence of severe periodontal disease, especially in younger individuals, can be a sign that warrants testing for HIV.

Dentists and healthcare providers can use the presence of periodontal disease as a diagnostic tool. If a patient presents with unexplained periodontal disease, especially if it is aggressive or atypical, it may prompt healthcare providers to consider HIV testing. For individuals already diagnosed with HIV, the severity and progression of periodontal disease can provide insights into the effectiveness of antiretroviral therapy and overall immune health. We documented in our study that dentition and periodontal status are statistically related to the presence of the HIV disease, as well as to the disease stage (CD4+ level and CD%). Recognizing and addressing periodontal issues early can facilitate timely HIV testing and intervention, ultimately improving patient outcomes.

However, this research is subject to several limitations. The first is related to the small number of patients included in the study that might not be representative of the population, potentially being at risk of bias. Second, as individuals under treatment with antiretroviral therapy were not included in the study, the findings might not represent this population group. Further studies are needed to evaluate the effect of antiretroviral therapy on periodontal diseases and their progress. Third, data regarding comorbidities that might impact the periodontal disease were gathered, even though the differences between groups were insignificant. Fourth, patients with incomplete data (first CD4+ and CD%) were excluded, influencing the number of patients included and likely the final results.

## Conclusions

HIV-infected patients manifest a worse dentition and periodontal status and the worsening was strongly correlated with the initial level of CD4+. Periodontal disease may serve as a significant clinical indicator for the early diagnosis of HIV and its progression. Dental professionals should be vigilant in assessing periodontal health, especially in high-risk populations, as it may prompt timely testing and intervention for HIV infection. Further research is needed to solidify these associations and improve the integration of dental care in HIV prevention and care strategies.

## Figures and Tables

**Figure 3:**
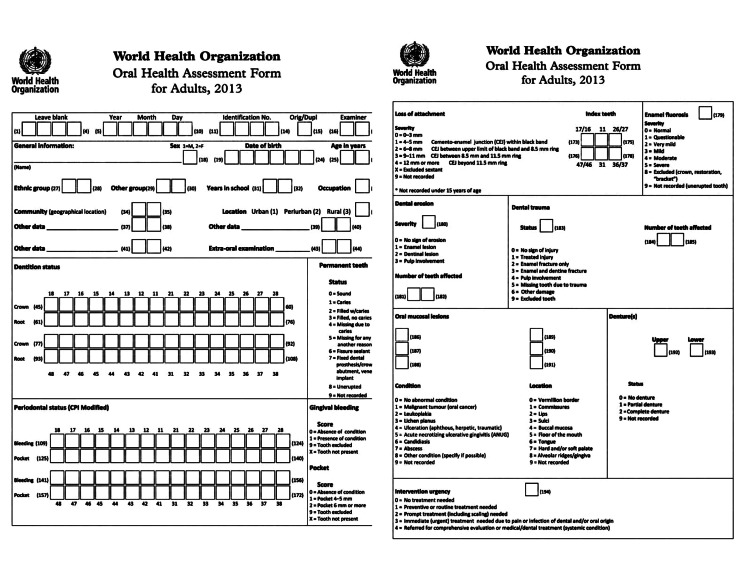
World Health Organization Oral Health Assessment Form for Adults, 2013.

**Figure 4:**
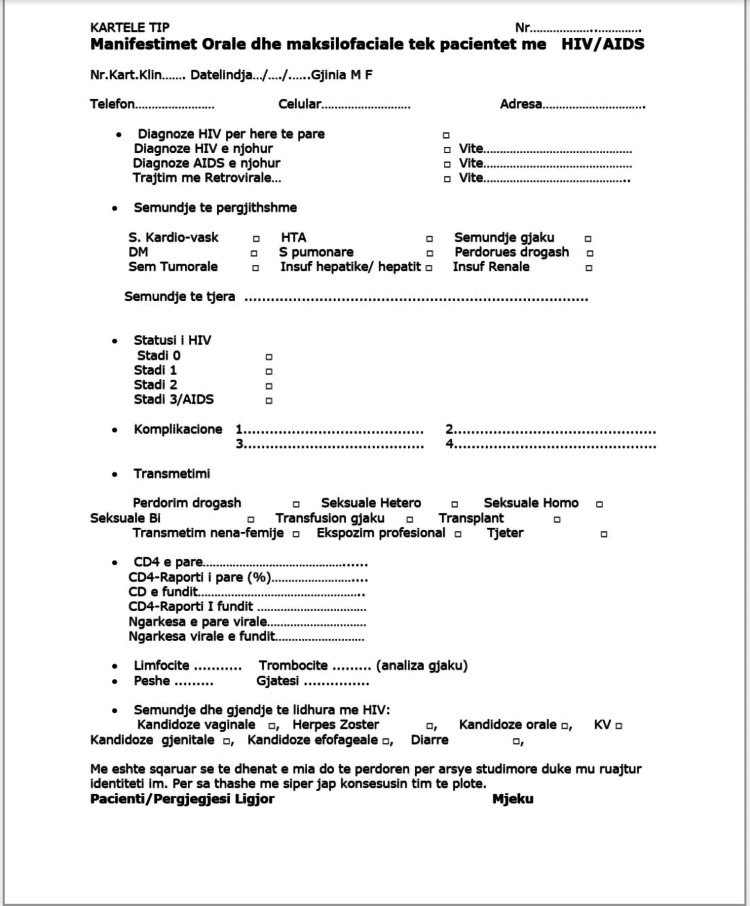
Questionnaire In Albanian.

**Figure 5:**
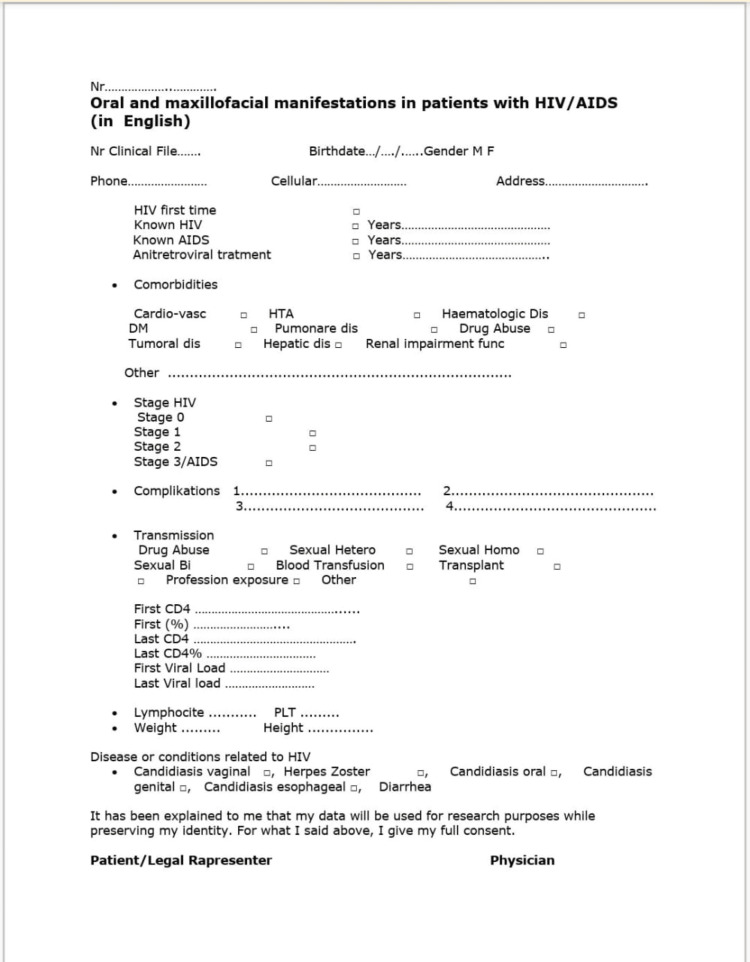
Questionnaire in English.
